# Supplementary material for: Monte Carlo simulation of OLS and linear mixed model inference of phenotypic effects on gene expression
Source: PeerJ. 2016 Oct 11;4:e2575. doi: 10.7717/peerj.2575 (PMC5068350; doi:10.7717/peerj.2575)
Supplement: Supplemental Information 1 — Supplemental tables 1 and 2 and R scripts for all analyses. [file peerj-04-2575-s001.pdf]

Monte Carlo simulation of OLS and linear mixed model inference of phenotypic effects on gene expression

Supplementary Material

Jeffrey A. Walker

September 14, 2016

1 Supplementary Tables

Table S1: Bootstrap standard errors of mean effects estimated by GLS. The bootstrap required excluding the variable *Smoke*. To show that the large difference between bootstrap and GLS standard errors is not due to this exclusion, the GLS estimates and standard errors with and without *Smoke* are given.

Type	Data	$\bar{\beta}$	SE	$\bar{\beta}$ (no smoke)	SE (no smoke)	SE_{boot}
Hedonia	FRED13	0.537	0.172	0.49	0.17	0.664
	FRED15	0.086	0.122	0.086	0.122	0.296
	FRED13+15	0.073	0.042	0.073	0.042	0.145
Eudaimonia	FRED13	0.135	0.177	0.174	0.173	0.66
	FRED15	-0.511	0.126	-0.511	0.126	0.349
	FRED13+15	-0.116	0.043	-0.118	0.043	0.25
$\delta_{hed-eud}$	FRED13	0.401	0.331	0.317	0.325	1.132
	FRED15	0.596	0.231	0.597	0.231	0.586
	FRED13+15	0.189	0.079	0.191	0.079	0.346

Table S2: Type I error, Power, Sign (S) error, and Exagerration Ratio (ER) for different levels of gene set size (m). This table is plotted in Fig. 3. Iter.I and Iter.II are the number of iterations in the Type I and Type II/S/M simulations. GA=Global Ancova, gee=Generalized Estimating Equations Wald test with robust SE, Obrien=Obrien's OLS test, permF= permutation under the null F test, R2=Anderson's permutation R_F^2 test, gls=GLS using heterogenous compound symmetry error matrix, gls.un=GLS using unstructured error matrix

model	n	m	Iter.I	TypeI	Iter.II	Power	S	ER
GA	122	10	4000	0.052	4000	0.31	0.109	1.7
gee	122	10	4000	0.083	4000	0.23	0.022	2.2
obrien	122	10	4000	0.05	4000	0.18	0.016	2.3
permF	122	10	4000	0.055	4000	0.31	0.11	1.7
R2	122	10	4000	0.051	4000	0.18	0.017	2.3
roast	122	10	4000	0.049	4000	0.17	0.016	2.3
gls	122	10	4000	0.103	4000	0.26	0.036	2.5
gls.un	122	10	2000	0.106	NA	NA	NA	NA
GA	122	30	4000	0.048	4000	0.44	0.097	1.5
gee	122	30	4000	0.075	4000	0.29	0.013	1.9
obrien	122	30	4000	0.047	4000	0.24	0.008	2
permF	122	30	4000	0.049	4000	0.43	0.099	1.5
R2	122	30	4000	0.048	4000	0.23	0.009	2.1
roast	122	30	4000	0.049	4000	0.23	0.011	2.1
gls	122	30	2000	0.164	2000	0.36	0.041	2.4
GA	122	52	4000	0.05	4000	0.48	0.093	1.4
gee	122	52	4000	0.078	4000	0.31	0.004	1.9
obrien	122	52	4000	0.047	4000	0.25	0.004	2
permF	122	52	4000	0.051	4000	0.47	0.093	1.4
R2	122	52	4000	0.047	4000	0.25	0.004	2
roast	122	52	4000	0.046	4000	0.24	0.004	2
gls	122	52	1000	0.279	1000	0.42	0.065	2.8

2 R Scripts

2.1 Main Script

```
# script Fredrickson_peerj.rev2.R to re-analyze Fredrickson et al 2013 and
# 2015 Jeffrey A. Walker August 22, 2016 clean version of
# Fredrickson_multivariate_lm.peerJ.rev1.R the scripts used for this
# manuscript are Fredrickson_peerj.rev2.R - scripts mostly specific to these
# datasets GSA_methods.R - generalized methods for any dataset GSA1.R - the
# Monte Carlo simulation of type I, II, S, M errors

library(data.table)
library(ggplot2)
library(reshape2)
library(GlobalAncova)  #bioconductor
library(globaltest)  #bioconductor
library(limma)  #bioconductor
library(mvtnorm)
library(nlme)
library(geepack)  #ditto
library(doBy)
library(showtext)  # needed for eps fonts to add Arial to .eps
font.add("Arial", regular = "Arial.ttf")
library(gridExtra)
library("grid")  # needed for 'unit' function in ggplot

# to install bioconductor packages use setRepositories() and choose '1 2'
# which is CRAN + BioC software
```

```r
# for Monte Carlo simulation of error rates run function start_here() in the
# script GSA1.R

do_Fredrickson <- function() {
    run_ols <- FALSE
    run_gee <- FALSE
    run_gls <- TRUE
    run_cole15 <- FALSE
    run_gls_parametric <- FALSE
    run_gls_bootstrap <- FALSE
    run_gls_permutation <- FALSE

    dt2013 <- get_cole_data(fn = "cole1_clean.txt", year = 2013, scale_it = TRUE)
    dt2015 <- get_cole_data(fn = "cole2_clean.txt", year = 2015, scale_it = TRUE)
    dt2015B <- get_cole_2015B(scale_it = TRUE)
    # combine the data illness in FRED2015 is averaged over the 13 categories so
    # divide illness in FRED13 by 13 to be in same scale as in FRED15. Even with
    # this the range of FRED15 is about 2X that of FRED13.
    # dt2013[,illness:=illness/13] # comment out to replicate FRED15 remove IL6
    # from dt2013
    redcols <- c(get_xcols(), c(pro_inflam_genes(year = 2015), antibody_genes(),
        ifn_genes()))
    dtCombi <- rbind(data.table(dt2013[, .SD, .SDcols = redcols], study = 2013),
        data.table(dt2015, study = 2015))  # use Fill=TRUE and full dt2015 data to retain IL6 to replicate FRE
    # dtCombi <-
    # rbind(data.table(dt2013,study=2013),data.table(dt2015,study=2015),fill=TRUE)
    # # use Fill=TRUE and full dt2015 data to retain IL6 to replicate FRED15
    dtCombi[, `:=`(study, factor(study))]

    ycols13 <- c(pro_inflam_genes(year = 2013), antibody_genes(), ifn_genes())
    ycols15 <- c(pro_inflam_genes(year = 2015), antibody_genes(), ifn_genes())
    xcols <- get_xcols()  # for all FRED datasets
    zcols <- c("zhedonia", "zeudaimonia")

    # do GLS and save so I don't have to keep doing this
    if (run_gls_parametric == TRUE) {
        saveRDS(gls_with_correlated_error(dt2013, xcols = xcols, ycols = ycols13,
            zcols = zcols, method = "gls"), "FRED13.gls.rds")
        saveRDS(gls_with_correlated_error(dt2015, xcols = xcols, ycols = ycols15,
            zcols = zcols, method = "gls"), "FRED15.gls.rds")
        saveRDS(gls_with_correlated_error(dtCombi, xcols = c(xcols, "study"),
            ycols = ycols15, zcols = zcols, method = "gls"), "FRED.Combi.gls.rds")
        # to replicate FRED15 saveRDS(gls_with_correlated_error(dtCombi,
        # xcols=c(xcols,'study'), ycols=ycols13, zcols=zcols, method='gls'),
        # 'FRED.Combi-rep.gls.rds')

        # redo 2013 and 2015 datasets without zhedonia to compare to COLE15
        xcolsb <- get_xcolsb()  # for COLE15
        xcols1 <- setdiff(xcols, "zhedonia")
        saveRDS(gls_with_correlated_error(dt2013, xcols = xcols1, ycols = ycols13,
            zcols = "zeudaimonia", method = "gls"), "FRED13.no-hedonia.gls.rds")
        saveRDS(gls_with_correlated_error(dt2015, xcols = xcols1, ycols = ycols15,
            zcols = "zeudaimonia", method = "gls"), "FRED15.no-hedonia.gls.rds")
        # COLE15 dropping 'hispanic' and 'ln_hh_income' # see original for more
        # variations
        saveRDS(gls_with_correlated_error(dt2015B[, .SD, .SDcols = setdiff(colnames(dt2015B),
            c("ln_hh_income", "hispanic"))], xcols = setdiff(xcolsb, c("ln_hh_income",
            "hispanic")), ycols = ycols13, zcols = "zeudaimonia", method = "gls"),
            "COLE15.gls.rds")
```

```
    # redo all three without smoke to see effect on standard error
    xcolsc <- setdiff(xcols, "smoke")
    saveRDS(gls_with_correlated_error(dt2013[, .SD, .SDcols = c(xcolsc,
        ycols13)], xcols = xcolsc, ycols = ycols13, zcols = zcols, method = "gls"),
        "FRED13-nosmoke.gls.rds")
    saveRDS(gls_with_correlated_error(dt2015[, .SD, .SDcols = c(xcolsc,
        ycols15)], xcols = xcolsc, ycols = ycols15, zcols = zcols, method = "gls"),
        "FRED15-nosmoke.gls.rds")
    saveRDS(gls_with_correlated_error(dtCombi[, .SD, .SDcols = c(xcolsc,
        "study", ycols15)], xcols = c(xcolsc, "study"), ycols = ycols15,
        zcols = zcols, method = "gls"), "FRED.Combi-nosmoke.gls.rds")

}

if (run_gls_bootstrap == TRUE) {
    xcolsc <- setdiff(xcols, "smoke")
    bootstrap_models(dt2013[, .SD, .SDcols = c(xcolsc, ycols13)], xcols = xcolsc,
        ycols = ycols13, zcols = zcols, which_file = "FRED13", tests = c("gls"),
        niter = 2)
    bootstrap_models(dt2015[, .SD, .SDcols = c(xcolsc, ycols15)], xcols = xcolsc,
        ycols = ycols15, zcols = zcols, which_file = "FRED15", tests = c("gls"),
        niter = 201)
    bootstrap_models(dtCombi[, .SD, .SDcols = c(xcolsc, "study", ycols15)],
        xcols = c(xcolsc, "study"), ycols = ycols15, zcols = zcols, which_file = "FRED.Combi",
        tests = c("gls"), niter = 201)
}

if (run_gls_permutation == TRUE) {
    permutation_gls(dt = dt2013, xcols = xcols, ycols = ycols13, zcols = zcols,
        method = "gls", perms = 200, write_it = TRUE, fn = "perm_gls.FRED13")
    permutation_gls(dt = dt2015, xcols = xcols, ycols = ycols15, zcols = zcols,
        method = "gls", perms = 200, write_it = TRUE, fn = "perm_gls.FRED15")
    permutation_gls(dt = dtCombi, xcols = c(xcols, "study"), ycols = ycols15,
        zcols = zcols, method = "gls", perms = 200, write_it = TRUE, fn = "perm_gls.FRED.Combi")
}

which_file_list <- c("FRED13", "FRED15", "FRED.Combi")
gls_table <- NULL
ols_table <- NULL
gee_table <- NULL
gls_supp_table <- NULL  # supplement

for (which_file in which_file_list) {
    covs <- xcols
    if (which_file == "FRED13") {
        dt <- copy(dt2013)
    }
    if (which_file == "FRED15") {
        dt <- copy(dt2015)
    }
    if (which_file == "FRED.Combi") {
        dt <- copy(dtCombi)
        covs <- c(xcols, "study")
    }
    if ("IL6" %in% colnames(dt)) {
        ycols <- ycols13
    } else {
        ycols <- ycols15
    }
```

```r
# some statistics on the gene expression levels
R <- cor(as.matrix(dt[, .SD, .SDcols = ycols]))
mean(abs(R[lower.tri(R)]))
max(abs(R[lower.tri(R)]))

# OLS table
if (run_ols == TRUE) {
    boot_mat <- ols.fit(dt, xcols = covs, ycols, zcols, perms = 10000,
        scale_it = TRUE, boot = TRUE, all = TRUE)
    boot_t <- make_ols_table(boot_mat)
    # obrien_t <-
    # rbind(obrien.fit(dt,xcols=covs,ycols,zcols='zhedonia'),obrien.fit(dt,xcols=covs,ycols,zcols='zeu
    # replace obrien_t with table computed including delta
    obrien_t <- obrien.fit.delta(dt, xcols = covs, ycols, zcols)
    perm_R2 <- permutation_t.fit(dt, xcols = covs, ycols, zcols, method = "R2",
        perms = 10000, write_it = FALSE, fn)
    perm_f <- permutation_F.fit(dt, xcols = covs, ycols, zcols, perms = 10000)
    ga <- run_GlobalAncova(dt, xcols = covs, ycols, zcols, perms = 10000)
    rot_z <- run_Roast(dt, xcols = covs, ycols, zcols = zcols, perms = 10000)

    # create NA for delta entry
    perm_R2 <- c(perm_R2, NA)
    perm_f <- c(perm_f, NA)
    ga <- c(ga, NA)

    # note that this assumes p-values are in same order as there is no checking
    # of row.names
    ols_table <- rbind(ols_table, data.table(Data = which_file, boot_t[,
        .(Type = Type, Estimate, SE_boot = SE)], SE_obrien = obrien_t[,
        SE], obrien = obrien_t[, prob], permR2 = perm_R2, ga = ga, permf = perm_f,
        roast = rot_z))
}

if (run_gee == TRUE) {
    # gee table
    fit <- gls_with_correlated_error(dt, xcols = covs, ycols, zcols,
        method = "gee")
    gee_res <- make_gls_table(fit, method = "gee")
    gee_part <- data.table(Type = row.names(gee_res), Data = which_file,
        gee_res[, c("Estimate", "Std.err", "Pr(>|W|)")])
    gee_part <- setNames(gee_part, c("Type", "Data", "Estimate", "SE",
        "p"))
    gee_table <- rbind(gee_table, gee_part)
}

if (run_gls == TRUE) {
    # gls table gls coefficients and p-values
    fit <- readRDS(paste(which_file, ".gls.rds", sep = ""))
    gls_res <- make_gls_table(fit, method = "gls")
    gls_part <- data.table(Type = row.names(gls_res), Data = which_file,
        gls_res[, c("Value", "Std.Error", "p-value")])
    gls_part <- setNames(gls_part, c("Type", "Data", "Estimate", "SE",
        "p"))

    # compute bootstrap.gls stats
    fn <- paste(which_file, ".bootstrap.gls.list.v2.txt", sep = "")
    gls_boot_res <- read_bootstrap.gls_list(fn)
    nrow(gls_boot_res)
    gls_boot_res[, `:=`(delta, b.zhedonia - b.zeudaimonia)]
```

```r
        boot.se <- apply(gls_boot_res[, .SD, .SDcols = c("b.zhedonia", "b.zeudaimonia",
            "delta")], 2, sd)
        gls_part <- cbind(gls_part, SE_boot = boot.se)

        # compute permutation.gls stats
        fn <- paste(which_file, ".permutation.gls.list.v1.txt", sep = "")
        gls_perm_res.v1 <- read_permutation.gls_list(fn)
        fn <- paste(which_file, ".permutation.gls.list.v2.txt", sep = "")
        gls_perm_res.v2 <- read_permutation.gls_list(fn)
        gls_perm_res.v2 <- convert_gls_permutation_to_old_format(gls_perm_res.v2)
        gls_perm_res <- gls_perm_res.v1  # v2 only to confirm new code
        nrow(gls_perm_res)
        gls_perm_p <- permutation_gls_p_value(gls_perm_res, statistic = "t")
        gls_part <- cbind(gls_part, perm_p = gls_perm_p)
        gls_table <- rbind(gls_table, gls_part)

        # supplement (boot) table
        fit <- readRDS(paste(which_file, "-nosmoke.gls.rds", sep = ""))
        gls_res_no_smoke <- data.table(make_gls_table(fit, method = "gls"))
        gls_no_smoke_part <- gls_part[, .(Type, Data, b = Estimate, SE)]
        gls_no_smoke_part <- cbind(gls_no_smoke_part, gls_res_no_smoke[,
            .(b.nosmoke = Value, SE.nosmoke = Std.Error)])
        gls_no_smoke_part <- cbind(gls_no_smoke_part, gls_part[, .(SE_boot)])
        gls_supp_table <- rbind(gls_supp_table, gls_no_smoke_part)

    }
}  # end which file


# clean tables

if (run_ols == TRUE) {
    ols_table_full <- copy(ols_table)
    write.table(ols_table_full, "ols_table_full.txt", quote = FALSE, row.names = FALSE,
        sep = "\t")
    ols_table <- data.table(read.table("ols_table_full.txt", header = TRUE,
        sep = "\t"))
    # make Type first column and drop SE_boot, which as the smoke problem
    ols_table <- ols_table[, .(Type, Data, Estimate, SE_obrien, obrien,
        permR2, ga, permf, roast)]
    ols_table[, `:=`(Type, factor(Type))]
    ols_table <- orderBy(~-Type, ols_table)
    ols_table[, `:=`(Estimate, round(Estimate, 3))]
    # ols_table[,SE_boot:=round(SE_boot,3)]
    ols_table[, `:=`(SE_obrien, round(SE_obrien, 3))]
    ols_table[, `:=`(obrien, round(obrien, 2))]
    ols_table[, `:=`(permR2, round(permR2, 2))]
    ols_table[, `:=`(ga, round(ga, 2))]
    ols_table[, `:=`(permf, round(permf, 2))]
    ols_table[, `:=`(roast, round(roast, 2))]
    # ols_table <- ols_table[Type!='delta'] # delta now in its own table
    write.table(ols_table, "ols_table.txt", quote = FALSE, row.names = FALSE,
        sep = "\t")
}

if (run_gls == TRUE) {
    gls_full_table <- copy(gls_table)
    gls_table[, `:=`(Type, factor(Type))]
    gls_table <- orderBy(~-Type, gls_table)
```

```r
        gls_table[, `:=`(Estimate, round(Estimate, 3))]
        gls_table[, `:=`(SE, round(SE, 3))]
        gls_table[, `:=`(p, round(p, 3))]
        gls_table[, `:=`(SE_boot, round(SE_boot, 3))]
        gls_table[, `:=`(perm_p, round(perm_p, 2))]
        write.table(gls_table, "gls_table.txt", quote = FALSE, row.names = FALSE,
            sep = "\t")

        # supplemental table
        gls_supp_table_full <- copy(gls_supp_table)
        gls_supp_table[, `:=`(Type, factor(Type))]
        gls_supp_table <- orderBy(~-Type, gls_supp_table)
        gls_supp_table[, `:=`(b, round(b, 3))]
        gls_supp_table[, `:=`(SE, round(SE, 3))]
        gls_supp_table[, `:=`(b.nosmoke, round(b.nosmoke, 3))]
        gls_supp_table[, `:=`(SE.nosmoke, round(SE.nosmoke, 3))]
        gls_supp_table[, `:=`(SE_boot, round(SE_boot, 3))]
        write.table(gls_supp_table, "gls_supp_table.txt", quote = FALSE, row.names = FALSE,
            sep = "\t")

    }

    if (run_gee == TRUE) {
        gee_table_full <- copy(gee_table)
        gee_table[, `:=`(Type, factor(Type))]
        gee_table <- orderBy(~-Type, gee_table)
        gee_table[, `:=`(Estimate, round(Estimate, 3))]
        gee_table[, `:=`(SE, round(SE, 3))]
        gee_table[, `:=`(p, round(p, 2))]
        write.table(gee_table, "gee_table.txt", quote = FALSE, row.names = FALSE,
            sep = "\t")
    }

    # make Cole15 table (table 2 in manuscript)
    if (run_cole15 == TRUE) {
        fit <- readRDS(paste("FRED13.no-hedonia.gls.rds"))
        cole15_table <- data.table(Data = "FRED13", t(summary(fit)$tTable["zeudaimonia",
            ]))
        fit <- readRDS(paste("FRED15.no-hedonia.gls.rds"))
        cole15_table <- rbind(cole15_table, data.table(Data = "FRED15", t(summary(fit)$tTable["zeudaimonia",
            ])))
        fit <- readRDS(paste("Cole15.gls.rds"))
        cole15_table <- rbind(cole15_table, data.table(Data = "COLE15", t(summary(fit)$tTable["zeudaimonia",
            ])))
        setnames(cole15_table, old = colnames(cole15_table), new = c("Data",
            "b.eudaimonia", "SE", "t", "p"))
        cole15_table <- cole15_table[, .(Data, b.eudaimonia, SE, p)]
        cole15_table[, `:=`(b.eudaimonia, round(b.eudaimonia, 3))]
        cole15_table[, `:=`(SE, round(SE, 3))]
        cole15_table[, `:=`(p, round(p, 3))]
        write.table(cole15_table, "cole15_table.txt", quote = FALSE, row.names = FALSE,
            sep = "\t")
    }

}

get_cole_data <- function(fn, year, scale_it = TRUE) {
    dt <- read_file(fn, year = year)
    dt[, `:=`(zhedonia, scale(zhedonia))]
```

```r
    dt[, `:=`(zeudaimonia, scale(zeudaimonia))]
    dt <- contrast_coefficients(dt)  # convert to CTRA response
    if (scale_it == TRUE) {
        xcols <- get_xcols()
        ycols <- c(pro_inflam_genes(year), antibody_genes(), ifn_genes())
        X <- dt[, .SD, .SDcols = xcols]
        Y <- scale(dt[, .SD, .SDcols = ycols])
        dt <- cbind(X, Y)
    }
    return(dt)
}

get_cole_2015B <- function(scale_it = TRUE) {
    fn <- "cole3_clean.txt"
    year <- 2013  #IL6 is present
    dt2015B <- data.table(read.table(fn, header = TRUE, sep = "\t"))
    dt2015B[, `:=`(female, factor(female))]
    dt2015B[, `:=`(black, factor(black))]
    dt2015B[, `:=`(smoke, factor(smoke))]
    dt2015B[, `:=`(hispanic, factor(hispanic))]
    dt2015B[, `:=`(alcohol, factor(alcohol))]
    xcolsb <- get_xcolsb()
    ycols <- c(pro_inflam_genes(year), antibody_genes(), ifn_genes())
    dt2015B <- na.omit(dt2015B[, .SD, .SDcols = c(xcolsb, ycols)])
    dt2015B[, `:=`(zeudaimonia, scale(zeudaimonia))]
    # note there is no zhedonia scale
    if (scale_it == TRUE) {
        X <- dt2015B[, .SD, .SDcols = xcolsb]
        Y <- scale(dt2015B[, .SD, .SDcols = ycols])
        dt2015B <- cbind(X, Y)
    }
    return(dt2015B)
}

read_file <- function(fn, year = 2013) {
    dt <- data.table(read.table(fn, header = TRUE, sep = "\t"))
    dt[, `:=`(male, factor(male))]
    dt[, `:=`(white, factor(white))]
    dt[, `:=`(smoke, factor(smoke))]
    xcols <- get_xcols()
    ycols <- c(pro_inflam_genes(year), antibody_genes(), ifn_genes())
    dt <- na.omit(dt[, .SD, .SDcols = c(xcols, ycols)])
    return(dt)
}

contrast_coefficients <- function(dt) {
    # dt is a matrix niter * p matrix of beta coefficients or raw data compute
    # contrast coefficients AND compute mean of these
    if ("IL6" %in% colnames(dt)) {
        year <- 2013
    } else {
        year <- 2015
    }
    ycols <- c(pro_inflam_genes(year), antibody_genes(), ifn_genes())
    rev_ycols <- c(antibody_genes(), ifn_genes())
    dt[, `:=`((rev_ycols), lapply(.SD, "*", -1)), .SDcols = rev_ycols]
    return(dt)
}
```

```r
get_xcols <- function() {
    # These are the regressors
    xcols <- c("male", "age", "white", "bmi", "alcohol", "smoke", "illness",
        "cd3d", "cd3e", "cd4", "cd8a", "fcgr3a", "cd19", "ncam1", "cd14", "zhedonia",
        "zeudaimonia")
    return(xcols)
}

get_xcolsb <- function() {
    xcolsb <- c("age", "female", "black", "smoke", "hispanic", "bmi", "diabcvdcastr",
        "ln_hh_income", "alcohol", "CD3D", "CD3E", "CD4", "CD8A", "FCGR3A",
        "CD19", "NCAM1", "CD14", "zeudaimonia")
    return(xcolsb)
}

# ycol functions

pro_inflam_genes <- function(year = 2013) {
    # from Frederickson 2013, note that 2015 does not include IL6 19
    # proinflammatory genes, which are up-regulated on average in the CTRA if
    # year=2013 include IL6, otherwise exclude it
    pro_inflam <- c("IL1A", "IL1B", "IL6", "IL8", "TNF", "PTGS1", "PTGS2", "FOS",
        "FOSB", "FOSL1", "FOSL2", "JUN", "JUNB", "JUND", "NFKB1", "NFKB2", "REL",
        "RELA", "RELB")
    if (year == 2015) {
        pro_inflam <- setdiff(pro_inflam, "IL6")
    }
    return(pro_inflam)
}

antibody_genes <- function() {
    # from Frederickson 2013, note that 2015 does not include IL6 three genes
    # involved in antibody synthesis, which are down-regulated on average in the
    # CTRA
    antibody <- c("IGJ", "IGLL1", "IGLL3")
    return(antibody)
}

ifn_genes <- function() {
    # from Frederickson 2013, note that 2015 does not include IL6 31 genes
    # involved in type I IFN responses, which are down-regulated on average in
    # the CTRA
    ifn <- c("GBP1", "IFI16", "IFI27", "IFI27L1", "IFI27L2", "IFI30", "IFI35",
        "IFI44", "IFI44L", "IFI6", "IFIH1", "IFIT1", "IFIT2", "IFIT3", "IFIT5",
        "IFIT1L", "IFITM1", "IFITM2", "IFITM3", "IFITM4P", "IFITM5", "IFNB1",
        "IRF2", "IRF7", "IRF8", "MX1", "MX2", "OAS1", "OAS2", "OAS3", "OASL")
    return(ifn)
}

obrien.fit.delta <- function(dt, xcols, ycols, zcols) {
    # This is obrien.fit from the GSA_methods.R scripts but I've added the
    # computation of the SE and p-value for the difference in effect between two
    # of the zcols (Hedonia and Eudaimonia)
    n <- nrow(dt)
    Y <- data.matrix(dt[, .SD, .SDcols = ycols])
    m <- length(ycols)
    p <- length(xcols)
    df <- n - p - 1
    b <- matrix(NA, nrow = 2, ncol = m)
```

```r
    se <- matrix(NA, nrow = 2, ncol = m)
    t_value <- matrix(NA, nrow = 2, ncol = m)
    sumR <- numeric(2)

    X.dm <- cbind(rep(1, n), data.matrix(dt[, .SD, .SDcols = xcols]))  # design matrix
    XTXI <- solve(t(X.dm) %*% X.dm)
    fit <- lm.fit(X.dm, Y)
    b <- fit$coefficients[zcols, ]
    e <- fit$residuals
    for (i in 1:length(zcols)) {
        Xred <- cbind(rep(1, n), data.matrix(dt[, .SD, .SDcols = setdiff(xcols,
            zcols[i])]))
        # Get residuals from X (so excluding zcols) to find R - the correlation
        # among the outcomes not explained by zcols
        fit <- lm.fit(Xred, Y)
        R <- cor(fit$residuals)
        sumR[i] <- sum(R)
        se[i, ] <- sqrt(diag((t(e) %*% e)/df) * XTXI[zcols[i], zcols[i]])
        t_value[i, ] <- b[i, ]/se[i, ]
    }

    # coef_table <- data.table(Estimate=b,se=se,t=t_value)
    obrien.b <- apply(b, 1, mean)
    obrien.t <- apply(t_value, 1, sum)/sqrt(sumR)
    obrien.sd <- obrien.b/obrien.t
    obrien.p <- 2 * pt(abs(obrien.t), df = df, lower.tail = FALSE)

    delta.b <- b[1, ] - b[2, ]
    delta.sd <- sqrt(se[1, ]^2 + se[2, ]^2)
    delta.t <- delta.b/delta.sd
    obrien.t.delta <- sum(delta.t)/sqrt(sumR[1] + sumR[2])
    p.delta <- 2 * pt(abs(obrien.t.delta), df = df, lower.tail = FALSE)
    obrien_table <- data.table(Type = c(zcols, "delta"), Estimate = c(obrien.b,
        mean(delta.b)), SE = c(obrien.sd, mean(delta.b)/obrien.t.delta), t = c(obrien.t,
        obrien.t.delta), prob = c(obrien.p, p.delta))
    return(obrien_table)
}



gls_with_correlated_error <- function(dt, xcols, ycols, zcols, method = "gls") {
    # generalized from original function to allow analysis of COLE15 dt is the
    # data in wide format xcols are the predictors ycols are the resposes zcols
    # are the focal predictors to return statistics
    dt[, `:=`(subject, factor(.I))]
    dtlong <- melt(dt, id.vars = c("subject", xcols), variable.name = "gene",
        value.name = "expression")
    dtlong[, `:=`(gene, factor(gene))]
    dtlong <- orderBy(~subject + gene, dtlong)
    form <- formula(paste("expression~", paste(c("gene", xcols), collapse = "+"),
        sep = ""))
    if (method == "gls") {
        fit1 <- gls(form, data = dtlong, method = "ML", correlation = corCompSymm(form = ~1 |
            subject), weights = varIdent(form = ~1 | gene), control = glsControl(msMaxIter = 500,
            msVerbose = FALSE), na.action = na.omit)
    }
    if (method == "lme") {
        fit1 <- lme(form, random = ~1 | subject, data = dtlong, method = "ML",
            correlation = corCompSymm(form = ~1 | subject), weights = varIdent(form = ~1 |
```

```
                gene), control = lmeControl(maxIter = 100, msMaxIter = 500,
                tolerance = 1e-06, msVerbose = FALSE))  # tolerance=1e-6 default
    }
    if (method == "gee") {
        fit1 <- geeglm(form, family = gaussian, data = dtlong, id = subject,
            waves = gene, corstr = "exchangeable", std.err = "san.se")
    }
    return(fit1)
}



bootstrap_models <- function(dt, xcols, ycols, zcols, which_file, tests = c("mv",
    "gls", "gee"), niter = 200, write_it = TRUE) {
    # bootstrap estimates using multivariate regression, glm, and gee models
    covs <- copy(xcols)
    if ("study" %in% colnames(dt)) {
        combi <- TRUE
    } else {
        combi <- FALSE
    }
    Y <- data.matrix(dt[, .SD, .SDcols = ycols])

    rows <- 1:nrow(dt)  # use if combi==FALSE
    # use if combi ==TRUE
    if (combi == TRUE) {
        rows1 <- which(dt[, study] == levels(dt[, study])[1])
        rows2 <- which(dt[, study] == levels(dt[, study])[2])
        s_rows1 <- copy(rows1)
        s_rows2 <- copy(rows2)
    }
    mv_matrix <- matrix(0, nrow = niter, ncol = 2)
    colnames(mv_matrix) <- zcols
    gee_table <- data.table(NULL)
    gls_table <- data.table(NULL)
    code <- paste(sample(LETTERS, 4, replace = TRUE), collapse = "")
    gee.out <- paste(which_file, code, "bootstrap.gee.table", "txt", sep = ".")
    gls.out <- paste(which_file, code, "bootstrap.gls.table", "txt", sep = ".")
    samp <- "obs"
    for (iter in 1:niter) {
        if (combi == FALSE) {
            X <- dt[rows, .SD, .SDcols = covs]
            Y <- scale(as.matrix(dt[rows, .SD, .SDcols = ycols]))
            dts <- cbind(X, Y)
            dts[, `:=`(zhedonia, scale(zhedonia))]
            dts[, `:=`(zeudaimonia, scale(zeudaimonia))]
        } else {
            dt1 <- dt[s_rows1, ]
            dt1[, `:=`(zhedonia, scale(zhedonia))]
            dt1[, `:=`(zeudaimonia, scale(zeudaimonia))]
            X1 <- dt1[, .SD, .SDcols = covs]
            Y1 <- scale(as.matrix(dt1[, .SD, .SDcols = ycols]))
            dt1 <- cbind(X1, Y1)

            dt2 <- dt[s_rows2, ]
            dt2[, `:=`(zhedonia, scale(zhedonia))]
            dt2[, `:=`(zeudaimonia, scale(zeudaimonia))]
            X2 <- dt2[, .SD, .SDcols = covs]
            Y2 <- scale(as.matrix(dt2[, .SD, .SDcols = ycols]))
```

```r
            dt2 <- cbind(X2, Y2)
            dts <- rbind(dt1, dt2)
        }

        if ("mv" %in% tests) {
            # not implemented in update
            Y.samp <- as.matrix(dts[, .SD, .SDcols = ycols])
            form <- formula(paste("Y.samp~", paste(xcols, collapse = "+"), sep = ""))
            fit <- lm(form, data = dt[rows, ])
            mv_matrix[iter, ] <- apply(coefficients(fit)[zcols, ], 1, mean)
        }
        if ("gls" %in% tests) {
            fit.gls <- gls_with_correlated_error(dts, xcols = covs, ycols = ycols,
                zcols = zcols, method = "gls")
            estimate <- summary(fit.gls)$tTable[zcols, "Value"]
            tvalue <- summary(fit.gls)$tTable[zcols, "t-value"]
            gls_table <- rbind(gls_table, data.table(samp = samp, b = t(estimate),
                t = t(tvalue)))
            if (write_it == TRUE) {
                write.table(gls_table, gls.out, quote = FALSE, row.names = FALSE,
                  sep = "\t")
            }
        }
        if ("gee" %in% tests) {
            fit.geeglm <- gls_with_correlated_error(dts, xcols = covs, ycols = ycols,
                zcols = zcols, method = "gee")
            # summary(lm(form,data=dtlong))£coefficients[zcols,]
            # summary(fit.geeglm)£coefficients[zcols,]
            estimate <- summary(fit.geeglm)$coefficients[zcols, "Estimate"]
            names(estimate) <- zcols
            gee_table <- rbind(gee_table, data.table(samp = samp, t(estimate)))
            if (write_it == TRUE) {
                write.table(gee_table, gee.out, quote = FALSE, row.names = FALSE,
                  sep = "\t")
            }
        }
        rows <- sample(1:nrow(dt), replace = TRUE)
        if (combi == TRUE) {
            s_rows1 <- sample(rows1, replace = TRUE)
            s_rows2 <- sample(rows2, replace = TRUE)
        }
        samp <- "resample"
    }
    return(NULL)
}

permutation_gls <- function(dt, xcols, ycols, zcols, method = "gls", perms = 200,
    write_it = FALSE, fn) {
    # uses Anderson permutation but fits with GLS dt is a data.table with the X
    # regressors and Y responses xcols are the regressors ycols are the
    # responses zcols are the responses that we care about method=GLS other
    # methods not available in this slimmed down version fn is the file name to
    # write to notes: the expected association between permuted hedonic score
    # and gene expression is zero so the expected delta is zero
    # E(E(b.h)-E(b.e))=0-0.

    code <- sample(LETTERS, 4, replace = TRUE)
    fn_full <- paste(fn, ".", paste(code, collapse = ""), ".txt", sep = "")
```

```
    p <- length(ycols)
    Y <- data.matrix(dt[, .SD, .SDcols = ycols])
    b_table <- data.table(NULL)
    # get residuals from covariates
    covs <- setdiff(xcols, zcols)
    X.full <- cbind(rep(1, nrow(dt)), data.matrix(dt[, .SD, .SDcols = xcols]))
    X.red <- cbind(rep(1, nrow(dt)), data.matrix(dt[, .SD, .SDcols = covs]))
    fit.obs <- lm.fit(X.red, Y)
    e <- fit.obs$residuals
    yhat <- fit.obs$fitted.values

    rows <- 1:nrow(dt)  # observed on first iter and permuted after
    samp <- "obs"
    for (iter in 1:perms) {
        Y.pi <- yhat + e[rows, ]  # permuted
        dts <- cbind(dt[, .SD, .SDcols = xcols], Y.pi)
        dts[, `:=`(subject, factor(.I))]  # need to recalc since dt is re-created
        dtlong <- melt(dts, id.vars = c("subject", xcols), variable.name = "gene",
            value.name = "expression")
        dtlong[, `:=`(gene, factor(gene))]
        dtlong <- orderBy(~subject + gene, dtlong)

        form <- formula(paste("expression~", paste(c("gene", xcols), collapse = "+"),
            sep = ""))
        if (method == "gls") {
            fit1 <- gls(form, data = dtlong, method = "ML", correlation = corCompSymm(form = ~1 |
                subject), weights = varIdent(form = ~1 | gene))
        }

        # save stats to table rbinding is slow but relative to the time it takes to
        # do the GLS calculation this is trivial also this works when there are two
        # variables in zcols.
        estimate <- summary(fit1)$tTable[zcols, "Value"]
        t_value = summary(fit1)$tTable[zcols, "t-value"]
        b_table <- rbind(b_table, data.table(permutation = samp, Ind.Var = zcols,
            Estimate = estimate, t.value = t_value))

        if (write_it == TRUE) {
            write.table(b_table, fn_full, quote = FALSE, sep = "\t", row.names = FALSE)
        }
        # permute rows
        rows <- sample(1:nrow(dt))
        samp <- "perm"
    }

    return(b_table)
}

run_GlobalAncova <- function(dt, xcols, ycols, zcols, perms = 10000) {
    prob <- NULL
    Y <- as.matrix(dt[, .SD, .SDcols = ycols])
    Yt <- t(Y)  # genes as rows

    form.full <- formula(paste("~", paste(xcols, collapse = "+"), sep = ""))
    model.dat <- dt[, .SD, .SDcols = xcols]
    for (i in 1:length(zcols)) {
        prob[zcols[i]] <- GlobalAncova(Yt, form.full, model.dat = model.dat,
            test.terms = zcols[i], method = "permutation", perm = perms)$test.result["p.perm",
            1]
```

```r
    }
    return(prob)
}

run_Roast <- function(dt, xcols, ycols, zcols, perms = 10000) {
    # Roast specific to the FRED datasets analyzing effects of zcols =
    # c('zhedonia', 'zeudaimonia')
    prob <- NULL
    Y <- as.matrix(dt[, .SD, .SDcols = ycols])
    Yt <- t(Y)  # genes as rows
    form <- formula(paste("~", paste(xcols, collapse = "+"), sep = ""))
    design <- model.matrix(form, data = dt)
    colnames(design)[1] <- "Intercept"  # change '(Intercept)' to 'Intercept'
    for (i in 1:length(zcols)) {
        Z <- which(colnames(design) == zcols[i])
        prob[zcols[i]] <- roast(y = Yt, design = design, contrast = Z, nrot = perms)$p["UpOrDown",
            "P.Value"]
    }
    cont.matrix <- makeContrasts(delta = "zhedonia-zeudaimonia", levels = design)
    prob["delta"] <- roast(y = Yt, design = design, contrast = cont.matrix,
        nrot = perms)$p["UpOrDown", "P.Value"]
    return(prob)
}

make_ols_table <- function(b_mat) {
    # returns ols estimate and bootstrap SE for parametric SE use Obrien?  if
    # all=TRUE then return matrix with rows=perms
    delta <- b_mat[, "zhedonia"] - b_mat[, "zeudaimonia"]
    b_mat <- cbind(b_mat, delta)
    b_table <- data.table(Type = colnames(b_mat), Estimate = b_mat[1, ], SE = apply(b_mat,
        2, sd))
    b_table[, `:=`(t, Estimate/SE)]
    b_table[, `:=`(prob, 2 * pt(abs(t), df = n - p - 1, lower.tail = FALSE))]  # conservative df, upper end sh
    return(b_table)
}

make_gls_table <- function(fit, method = "gls") {
    zcols = c("zhedonia", "zeudaimonia")
    if (method == "gls") {
        gls_table <- summary(fit)$tTable[zcols, ]
    }
    if (method == "gee") {
        gls_table <- summary(fit)$coefficients[zcols, ]
    }
    # get delta
    coef_names <- names(fit$coefficients)
    hed_i <- which(coef_names == "zhedonia")
    eud_i <- which(coef_names == "zeudaimonia")
    p <- length(coef_names)
    lambda <- numeric(p)
    lambda[hed_i] <- 1
    lambda[eud_i] <- -1
    delta_row <- esticon(fit, lambda)[, c("Estimate", "Std.Error", "X2.value",
        "Pr(>|X^2|)")]
    row.names(delta_row)[1] <- "delta"
    colnames(delta_row) <- colnames(gls_table)
    gls_table <- rbind(gls_table, delta_row)

    return(gls_table)
```

```
}

read_bootstrap.gls_list <- function(fn) {
    # this is the old format
    the_list <- as.character(read.table(fn)[, 1])
    dt <- data.table(NULL)
    for (i in 1:length(the_list)) {
        file_name <- the_list[i]
        part_dt <- data.table(read.table(file_name, header = TRUE))
        if (i > 1) {
            # exclude rows with 'obs'
            inc <- which(part_dt[, samp != "obs"])
            part_dt <- part_dt[inc]
        }
        dt <- rbind(dt, part_dt)
    }
    return(dt)
}

read_permutation.gls_list <- function(fn) {
    the_list <- as.character(read.table(fn)[, 1])
    dt <- data.table(NULL)
    for (i in 1:length(the_list)) {
        file_name <- the_list[i]
        part_dt <- data.table(read.table(file_name, header = TRUE))
        if (i > 1) {
            # exclude rows with 'obs'
            inc <- which(part_dt[, permutation != "obs"])
            part_dt <- part_dt[inc]
        }
        dt <- rbind(dt, part_dt)
    }
    return(dt)
}

convert_gls_permutation_to_old_format <- function(long) {
    # long is the data.table in the new format
    zhedonia <- long[Ind.Var == "zhedonia"]
    zeudaimonia <- long[Ind.Var == "zeudaimonia"]
    dt <- cbind(zhedonia[, .(permutation, coeff.zhedonia = Estimate, t.zhedonia = t.value)],
        zeudaimonia[, .(coeff.zeudaimonia = Estimate, t.zeudaimonia = t.value)])
    return(dt)
}

permutation_gls_p_value <- function(res, statistic = "t") {
    if (statistic == "t") {
        inc <- which(substr(colnames(res), 1, 1) == "t")
        # get p-value for delta
        t.value <- res[, .SD, .SDcols = colnames(res)[inc]]
        inc <- which(substr(colnames(res), 1, 1) == "c")
        coeff.value <- res[, .SD, .SDcols = colnames(res)[inc]]
        # delta.se <- sqrt(se[Type=='hedonic',se]^2 + se[Type=='eudaimonic',se]^2)
        se.value <- coeff.value/t.value
        delta.se <- sqrt(apply(se.value^2, 1, sum))
        delta.t <- (coeff.value[, coeff.zhedonia] - coeff.value[, coeff.zeudaimonia])/delta.se
        t.value <- cbind(t.value, delta = delta.t)
        p.value <- apply(abs(t.value), 2, function(x) length(which(x >= x[1]))/length(x))
    }
    if (statistic == "c") {
```

```
        inc <- which(substr(colnames(res), 1, 1) == "c")
        p.value <- apply(abs(res[, .SD, .SDcols = colnames(res)[inc]]), 2, permutation.p.value)
        p.value <- c(p.value, delta = permutation.p.value(abs(res[, coeff.zhedonia] -
            res[, coeff.zeudaimonia])))
    }
    return(p.value)
}

permutation_gls_p_value.v2 <- function(res, zcols, statistic = "t") {
    # new formatting of input file
    prob <- NULL
    if (statistic == "t") {
        for (iv in zcols) {
            t <- res[Ind.Var == iv, t.value]
            prob[iv] <- length(which(abs(t) >= abs(t[1])))/length(t) * 100
        }
        # delta
    }
    if (statistic == "c") {
    }
    return(prob)
}

figure_1 <- function() {
    # residual vs. fitted for GLS and GEE
    dt <- copy(dt2015)
    xcols <- get_xcols()
    ycols <- ycols15
    zcols <- c("zhedonia", "zeudaimonia")
    fit.gls <- readRDS(paste("FRED15", ".gls.rds", sep = ""))
    fit.gee <- gls_with_correlated_error(dt, xcols = xcols, ycols, zcols, method = "gee")
    # gls residuals vs. fitted
    qplot(x = fitted(fit.gls), y = residuals(fit.gls))
    qplot(x = fit.gee$fitted.values, y = fit.gee$residuals)

    dt.gls <- data.table(fitted = fitted(fit.gls), residuals = residuals(fit.gls))
    gg1 <- ggplot(data = dt.gls, aes(x = fitted, y = residuals))
    gg1 <- gg1 + geom_point()
    gg1 <- gg1 + labs(x = "Fitted", y = "Residuals")
    gg1 <- gg1 + ggtitle("A")
    gg1 <- gg1 + theme_bw() + theme(axis.title = element_text(size = 10), axis.text = element_text(size = 8),
        plot.title = element_text(hjust = 0), strip.text = element_text(size = 8),
        legend.title = element_blank(), legend.position = c(0.26, 0.16), plot.margin = unit(x = c(0,
            0.1, 0, 0), "cm"))
    gg1

    dt.gee <- data.table(fitted = fit.gee$fitted.values, residuals = fit.gee$residuals)
    setnames(dt.gee, old = colnames(dt.gee), new = colnames(dt.gls))
    gg2 <- ggplot(data = dt.gee, aes(x = fitted, y = residuals))
    gg2 <- gg2 + geom_point()
    gg2 <- gg2 + labs(x = "Fitted", y = "Residuals")
    gg2 <- gg2 + ggtitle("B")
    gg2 <- gg2 + theme_bw() + theme(axis.title = element_text(size = 10), axis.text = element_text(size = 8),
        plot.title = element_text(hjust = 0), strip.text = element_text(size = 8),
        legend.title = element_blank(), legend.position = c(0.26, 0.16), plot.margin = unit(x = c(0,
            0.1, 0, 0), "cm"))
    gg2

    fig_name <- paste("Fig_1_diagnostics.pdf", sep = "")
```

```r
    pdf(fig_name, paper = "special", onefile = FALSE, width = 6.5, height = 3)
    # postscript('fig_01.eps',horizontal=FALSE,onefile=FALSE,paper='special',height=3,width=6.5)
    showtext.begin()
    print(gg1)
    print(gg2)
    grid.arrange(gg1, gg2, ncol = 2, nrow = 1)
    showtext.end()
    dev.off()

}

figure_2 <- function() {
    # A scatterplot of regression coefficients for x=hedonia y=eudaimonia for
    # GLS bootstrap
    zcols <- c("zhedonia", "zeudaimonia")
    # bootstrap gls
    fn <- paste("FRED15.bootstrap.gls.list.v2.txt", sep = "")
    gls_boot <- read_bootstrap.gls_list(fn)
    # replace obs with the .nosmoke results since these are what is bootstrapped
    obs <- summary(readRDS(paste("FRED15", "-nosmoke.gls.rds", sep = "")))$tTable[zcols,
        "Value"]
    gls_boot[samp == "obs", `:=`(b.zhedonia, obs[["zhedonia"]])]
    gls_boot[samp == "obs", `:=`(b.zeudaimonia, obs[["zeudaimonia"]])]
    # limit to iter=200
    if (nrow(gls_boot) > 200) {
        gls_boot <- gls_boot[1:200, ]
    }
    # get standard errors and CI
    apply(gls_boot[, .SD, .SDcols = c("b.zhedonia", "b.zeudaimonia")], 2, sd)
    apply(gls_boot[, .SD, .SDcols = c("b.zhedonia", "b.zeudaimonia")], 2, quantile,
        probs = c(0.025, 0.975))
    gls_boot[, `:=`(color, factor(ifelse(samp == "resample", 0, 1)))]
    gls_boot <- orderBy(~color, data = gls_boot)
    r <- cor(gls_boot[, b.zhedonia], gls_boot[, b.zeudaimonia])
    gg <- ggplot(data = gls_boot, aes(x = b.zhedonia, y = b.zeudaimonia, color = color))
    gg <- gg + geom_point(size = 2)
    gg <- gg + scale_colour_manual(values = c("grey", "black"), labels = c("Resampled",
        "Observed"))
    gg <- gg + labs(x = "Hedonia", y = "Eudaimonia")
    gg <- gg + theme_bw() + theme(axis.title = element_text(size = 10), axis.text = element_text(size = 8),
        plot.title = element_text(hjust = 0), strip.text = element_text(size = 8),
        legend.title = element_blank(), legend.position = c(0.26, 0.16), plot.margin = unit(x = c(0,
            0.1, 0, 0), "cm"))
    gg
    ggExtra::ggMarginal(gg, type = "histogram")
    gg1 <- gg

    fig_name <- paste("Fig_2_gls_boot.pdf", sep = "")
    pdf(fig_name, paper = "special", onefile = FALSE, width = 3, height = 3)
    # postscript('fig_01.eps',horizontal=FALSE,onefile=FALSE,paper='special',height=3,width=6.5)
    showtext.begin()
    print(gg1)
    ggExtra::ggMarginal(gg1, type = "histogram")
    showtext.end()
    dev.off()

}

# figure_3 is computed in the GSA1.R script
```

```
figure_4 <- function() {
    # bivariate plot of permutation gls effects
    fn <- paste("FRED15", ".permutation.gls.list.v1.txt", sep = "")
    gls_perm_res <- read_permutation.gls_list(fn)
    gls_perm_res[, `:=`(color, factor(ifelse(permutation == "perm", 0, 1)))]
    # gls_boot <-
    # rbind(gls_boot,data.table(samp='FRED13',b.zhedonia=fred13['zhedonia'],b.zeudaimonia=fred13['zeudaimonia'
    gls_perm_res <- orderBy(~color, data = gls_perm_res)

    # compare 95% tile
    apply(gls_mc[model == "gls", .SD, .SDcols = c("b", "b.hed")], 2, quantile,
        prob = c(0.025, 0.975))
    apply(gls_perm_res[, .SD, .SDcols = c("coeff.zeudaimonia", "coeff.zhedonia")],
        2, quantile, prob = c(0.025, 0.975))


    gg <- ggplot(data = gls_perm_res, aes(x = coeff.zhedonia, y = coeff.zeudaimonia,
        color = color))
    gg <- gg + geom_point(size = 2)
    gg <- gg + scale_colour_manual(values = c("grey", "black"), labels = c("Permuted",
        "Observed"))
    gg <- gg + labs(x = "Hedonia", y = "Eudaimonia")
    # gg <- gg + ggtitle('B')
    gg <- gg + theme_bw() + theme(axis.title = element_text(size = 10), axis.text = element_text(size = 8),
        plot.title = element_text(hjust = 0), strip.text = element_text(size = 8),
        legend.title = element_blank(), legend.position = c(0.26, 0.16), plot.margin = unit(x = c(0,
            0.1, 0, 0), "cm"))
    gg
    gg2 <- gg


    fig_name <- paste("Fig_4_gls_sim.pdf", sep = "")
    pdf(fig_name, paper = "special", onefile = FALSE, width = 3, height = 3)
    # postscript('fig_01.eps',horizontal=FALSE,onefile=FALSE,paper='special',height=3,width=6.5)
    showtext.begin()
    print(gg2)
    ggExtra::ggMarginal(gg2, type = "histogram")
    showtext.end()
    dev.off()

}
```

2.2 Monte Carlo Simulation Script

```
# Script GSA1.R
# Scripts for Monte Carlo simulation of error rates of GSA methods applied to model of
# Fredrickson et al 2015 data
# Jeffrey A. Walker
# August, 22, 2016
# cleaning of code from Fredrickson_multivariate_lm.peerj.rev1
# Monte Carlo results for manuscript re-run using this and not original code
# requires functions in
  # Fredrickson_peerj.rev2.R
  # GSA_methods.R

library(data.table)
library(ggplot2)
```

```
library(reshape2)
library(GlobalAncova) #bioconductor
library(globaltest) #bioconductor
library(limma) #bioconductor
library(mvtnorm)
library(nlme)
library(geepack) #ditto
library(doBy)
library(showtext) # needed for eps fonts to add Arial to .eps
font.add('Arial',regular='Arial.ttf')
library(gridExtra)
library("grid") # needed for "unit" function in ggplot

# to install bioconductor packages use
# setRepositories()
# and choose "1 2" which is CRAN + BioC software

start_here <- function(){
  #do_it()
  res_table <- table_error_rates()
  error_table <- clean_error_table(copy(res_table))
  print(error_table)
  error_figure(res_table[model!='gls.un',])
  write.table(error_table,'error_table.txt',row.names=FALSE,sep='\t',quote=FALSE)
}

do_it <- function(){
  # this takes about 48 hours on my macbook
  method_list <- c('R2','permF','roast','GA','obrien','gee')
  niter <- 4000
  perms <- 2000
  m_array <- c(10,30,52)
  for(m in m_array){
    res1 <- simulate_it_1(niter=niter,beta=-9999,method_list=method_list,n_array=-9999, m_array=m, perms=perms
    res2 <- simulate_it_1(niter=niter,beta=-9999,method_list=method_list,n_array=-9999, m_array=m, perms=perms
  }

  # Because of the time to model GLS, GLS was run separately and on multiple computers using
  # versions of this
  method_list <- c('gls') # office macs
  niter <- 200 # 4000, 2000, 1000 for m = 10, 30, 52
  perms <- 2000
  m_array <- c(52)
  for(m in m_array){
    res1 <- simulate_it_1(niter=niter,beta=-9999,method_list=method_list,n_array=-9999, m_array=m, perms=perms
    #res2 <- simulate_it_1(niter=niter,beta=-9999,method_list=method_list,n_array=-9999, m_array=m, perms=perm
  }

  # an attempt to run an unstructured matrix
  method_list <- c('gls.un') # office macs
  niter <- 200 # 4000, 2000, 1000 for m = 10, 30, 52
  perms <- 2000
  m_array <- c(52)
  for(m in m_array){
    #res1 <- simulate_it_1(niter=niter,beta=-9999,method_list=method_list,n_array=-9999, m_array=m, perms=perm
    res2 <- simulate_it_1(niter=niter,beta=-9999,method_list=method_list,n_array=-9999, m_array=m, perms=perms
  }
```

```
}


table_error_rates <- function(){ # and Figure!
  # script to collect the results files of the simulation in function do_it and
  # table the error types and generate the figure for the manuscript

  res1 <- data.table(NULL)
  res1 <- rbind(res1,data.table(read.table('gsa_sim_1.typeI.WOUI.txt',header=TRUE,sep='\t')))
  res1 <- rbind(res1,data.table(read.table('gsa_sim_1.typeI.JKKC.txt',header=TRUE,sep='\t')))
  res1 <- rbind(res1,data.table(read.table('gsa_sim_1.typeI.SMNX.txt',header=TRUE,sep='\t')))
  res1 <- rbind(res1,data.table(read.table('gsa_sim_1.typeI.BILN.txt',header=TRUE,sep='\t')))
  res1 <- rbind(res1,data.table(read.table('gsa_sim_1.typeI.BMRD.txt',header=TRUE,sep='\t')))
  res1 <- rbind(res1,data.table(read.table('gsa_sim_1.typeI.HQRH.txt',header=TRUE,sep='\t')))
  res1 <- rbind(res1,data.table(read.table('gsa_sim_1.typeI.LZAH.txt',header=TRUE,sep='\t')))
  res1 <- rbind(res1,data.table(read.table('gsa_sim_1.typeI.GALD.txt',header=TRUE,sep='\t')))
  res1 <- rbind(res1,data.table(read.table('gsa_sim_1.typeI.ROGL.txt',header=TRUE,sep='\t')))
  res1 <- rbind(res1,data.table(read.table('gsa_sim_1.typeI.IPXZ.txt',header=TRUE,sep='\t')))
  res1 <- rbind(res1,data.table(read.table('gsa_sim_1.typeI.RPYK.txt',header=TRUE,sep='\t')))
  res1 <- rbind(res1,data.table(read.table('gsa_sim_1.typeI.QGZB.txt',header=TRUE,sep='\t')))
  res1 <- rbind(res1,data.table(read.table('gsa_sim_1.typeI.NMYM.txt',header=TRUE,sep='\t')))
  res1 <- rbind(res1,data.table(read.table('gsa_sim_1.typeI.QCWH.txt',header=TRUE,sep='\t')))
  res1 <- rbind(res1,data.table(read.table('gsa_sim_1.typeI.LGIX.txt',header=TRUE,sep='\t')))
  res1 <- rbind(res1,data.table(read.table('gsa_sim_1.typeI.ETOD.txt',header=TRUE,sep='\t')))

  res2 <- data.table(NULL)
  res2 <- rbind(res2,data.table(read.table('gsa_sim_1.typeII.YOGT.txt',header=TRUE,sep='\t')))
  res2 <- rbind(res2,data.table(read.table('gsa_sim_1.typeII.RPCV.txt',header=TRUE,sep='\t')))
  res2 <- rbind(res2,data.table(read.table('gsa_sim_1.typeII.ELSO.txt',header=TRUE,sep='\t')))
  res2 <- rbind(res2,data.table(read.table('gsa_sim_1.typeII.DTEO.txt',header=TRUE,sep='\t')))
  res2 <- rbind(res2,data.table(read.table('gsa_sim_1.typeII.UULC.txt',header=TRUE,sep='\t')))
  res2 <- rbind(res2,data.table(read.table('gsa_sim_1.typeII.DMYB.txt',header=TRUE,sep='\t')))
  res2 <- rbind(res2,data.table(read.table('gsa_sim_1.typeII.ONKA.txt',header=TRUE,sep='\t')))
  res2 <- rbind(res2,data.table(read.table('gsa_sim_1.typeII.HFZQ.txt',header=TRUE,sep='\t')))
  res2 <- rbind(res2,data.table(read.table('gsa_sim_1.typeII.XGGD.txt',header=TRUE,sep='\t')))
  res2 <- rbind(res2,data.table(read.table('gsa_sim_1.typeII.IPZD.txt',header=TRUE,sep='\t')))
  res2 <- rbind(res2,data.table(read.table('gsa_sim_1.typeII.UWHY.txt',header=TRUE,sep='\t')))
  res2 <- rbind(res2,data.table(read.table('gsa_sim_1.typeII.NRNJ.txt',header=TRUE,sep='\t')))
  res2 <- rbind(res2,data.table(read.table('gsa_sim_1.typeII.AMOB.txt',header=TRUE,sep='\t')))

  # use only first 1000 rows of gls m=52
  inc <- which(res1[,model]=='gls' & res1[,m]==52)
  diffinc <- setdiff(1:nrow(res1),inc)
  part1 <- res1[diffinc]
  keep <- min(1000,length(inc))
  part2 <- res1[inc[1:keep]]
  res1 <- rbind(part1,part2)

  inc <- which(res2[,model]=='gls' & res2[,m]==52)
  diffinc <- setdiff(1:nrow(res2),inc)
  part1 <- res2[diffinc]
  keep <- min(1000,length(inc))
  part2 <- res2[inc[1:keep]]
  res2 <- rbind(part1,part2)

  alpha <- 0.05
  trueb <- 0.06716244 # the value of beta used in the simulation with an effect
  t1 <- res1[,.(N.I=.N,TypeI=length(which(prob<=alpha))/.N),by=.(model,n,m)] #MAE = mean absolute error
  # for type II
```

```
  t2 <- res2[,.(N.II=.N,Power=length(which(prob<=alpha & b>0))/.N, S=length(which(prob<=alpha & b<0))/length(w
  t3 <- res2[prob<=alpha & b>0,.(ER=mean(b/trueb)),by=.(model,n,m)]

  res_table <- merge(t1,t2,by=c('model','n','m'),all=TRUE)
  res_table <- merge(res_table,t3,by=c('model','n','m'),all=TRUE)
  res_table <- orderBy(~n + m, data=res_table)
  #res_table

  # compute bias
  res_table[m==52,.(res_table[m==52 & model=='GA',Power]/Power),by=model]
  t4 <- res2[,.(b_hat=round((mean(b)-trueb)/trueb*100,4)),by=.(model,n,m)] # test for bias
  return(res_table)

}

gls_adjusted_stats <- function(res_table){
  # ust this to find the adjusted alpha to make the GLS type I error == 0.05 to see how this affects power
  alpha <- 0.00015
  t1 <- res1[,.(N.I=.N,TypeI=length(which(prob<=alpha))/.N),by=.(model,n,m)] #MAE = mean absolute error
  t2 <- res2[,.(N.II=.N,Power=length(which(prob<=alpha & b>0))/.N, S=length(which(prob<=alpha & b<0))/length(w
  t3 <- res2[prob<=alpha & b>0,.(ER=mean(b/trueb)),by=.(model,n,m)]

  alt_table <- merge(t1,t2,by=c('model','n','m'),all=TRUE)
  alt_table <- merge(alt_table,t3,by=c('model','n','m'),all=TRUE)
  alt_table <- orderBy(~n + m, data=alt_table)
  alt_table[model=='gls']
  # for type II
  # how much power does GLS have when type I = 0.05?
  #m=10, alpha = 0.0155, GLS type I = 0.05000, Power = 0.156500
  #m=30, alpha = 0.0037, GLS type I = 0.05000, Power = 0.1445000
  #m=52, alpha = 0.00015, GLS type I = 0.05000, Power = 0.11500
}

clean_error_table <- function(error_table){
  # input is res_table
  error_table[,TypeI:=round(TypeI,3)]
  error_table[,Power:=round(Power,2)]
  error_table[,S:=round(S,3)]
  error_table[,ER:=round(ER,1)]
  return(error_table)
}

error_figure <- function(res_table){
  # res_table is the output from
  gg <- ggplot(data=res_table,aes(x=m,y=TypeI,color=model))
  gg <- gg + geom_point(aes(shape=model))
  gg <- gg + scale_shape_manual(values=c(0,1,2,5,16,17,15))
  gg <- gg + geom_line()
  gg <- gg + ggtitle('A')
  gg <- gg + labs(x = 'genes',y = 'Type I')
  gg <- gg + scale_colour_brewer(palette = "Dark2")
  gg <- gg + theme_bw() + theme(axis.title=element_text(size=10),axis.text=element_text(size=8),plot.title=eler
  gg1 <- gg
  gg

  gg <- ggplot(data=res_table,aes(x=m,y=Power,color=model))
  gg <- gg + geom_point(aes(shape=model))
  gg <- gg + scale_shape_manual(values=c(0,1,2,5,16,17,15))
  gg <- gg + geom_line()
```

```
gg <- gg + ggtitle('B')
gg <- gg + labs(x = 'genes')
gg <- gg + scale_colour_brewer(palette = "Dark2")
gg <- gg + theme_bw() + theme(axis.title=element_text(size=10),axis.text=element_text(size=8),plot.title=ele
gg2 <- gg
gg

gg <- ggplot(data=res_table,aes(x=m,y=S,color=model))
gg <- gg + geom_point(aes(shape=model))
gg <- gg + scale_shape_manual(values=c(0,1,2,5,16,17,15))
gg <- gg + geom_line()
gg <- gg + ggtitle('C')
gg <- gg + labs(x = 'genes',y = 'Type S')
gg <- gg + scale_colour_brewer(palette = "Dark2")
gg <- gg + theme_bw() + theme(axis.title=element_text(size=10),axis.text=element_text(size=8),plot.title=ele
gg3 <- gg
gg

gg <- ggplot(data=res_table,aes(x=m,y=ER,color=model))
gg <- gg + geom_point(aes(shape=model))
gg <- gg + scale_shape_manual(values=c(0,1,2,5,16,17,15))
gg <- gg + geom_line()
gg <- gg + ggtitle('D')
gg <- gg + labs(x = 'genes',y = 'Exaggeration Ratio')
gg <- gg + scale_colour_brewer(palette = "Dark2")
gg <- gg + theme_bw() + theme(axis.title=element_text(size=10),axis.text=element_text(size=8),plot.title=ele
gg4 <- gg
gg

fig_name <- paste('Fig_errors.pdf',sep='')
pdf(fig_name,paper='special',onefile=FALSE,width=5.5,height=5.5)
#  postscript('fig_01.eps',horizontal=FALSE,onefile=FALSE,paper='special',height=3,width=6.5)
showtext.begin()
print(gg1)
print(gg2)
print(gg3)
print(gg4)
grid.arrange(gg1,gg2,gg3,gg4,ncol=2,nrow=2)
showtext.end()
dev.off()

# print a legend
gg <- ggplot(data=res_table,aes(x=m,y=ER,color=model, shape=model))
gg <- gg + geom_point()
gg <- gg + geom_line()
gg <- gg + ggtitle('D')
gg <- gg + labs(y = 'Exaggeration Ratio')
gg <- gg + scale_colour_brewer(palette = "Dark2",labels=c('Fga','gee','obrien','Fpun','R2','roast','gls'))
gg <- gg + scale_shape_manual(values=c(0,1,2,5,16,17,15),labels=c('Fga','gee','obrien','Fpun','R2','roast','
gg <- gg + theme_bw() + theme(axis.title=element_text(size=10),axis.text=element_text(size=8),plot.title=ele
#gg <- gg + guides(color = guide_legend(label.position = "bottom"))
gg

legend <- ggplot_gtable(ggplot_build(gg))$grobs
#dev.new()
#pushViewport(plotViewport(rep(1, 4)))
#grid.draw(legend[[8]])
```

```
  fig_name <- paste('Fig_errors_legend.pdf',sep='')
  pdf(fig_name,paper='special',onefile=FALSE,width=4.75,height=.5)
  #  postscript('fig_01.eps',horizontal=FALSE,onefile=FALSE,paper='special',height=3,width=6.5)
  showtext.begin()
  grid.draw(legend[[8]])
  showtext.end()
  dev.off()


}

bias_plot <- function(){
  # need res2 from table_error_rates
  trueb <- 0.06716244 # the value of beta used in the simulation with an effect
  olsdata <- res2[model=='obrien' & m==52,]
  glsdata <- res2[model=='gls' & m==52,]
  N.ols <- nrow(olsdata)
  N.gls <- nrow(glsdata)
  niter <- 10000
  iters <- as.integer(runif(niter,100,N.gls))
  b.ols <- matrix(0,nrow=niter,ncol=2)
  colnames(b.ols) <- c('iters','b')
  b.gls <- copy(b.ols)
  for(iter in 1:niter){
    n <- iters[iter] # number of rows to sample
    inc <- sample(1:N.ols,n)
    b.ols[iter,] <- c(n,mean(olsdata[inc,b]))
    inc <- sample(1:N.gls,n)
    b.gls[iter,] <- c(n,mean(glsdata[inc,b]))
  }
  dt <- rbind(data.table(method='ols',b.ols),data.table(method='gls',b.gls))
  gg <- ggplot(data=dt,aes(x=iters,y=b,color=method))
  gg <- gg + geom_point(alpha = .1)
  gg

  #bias
  (mean(b.gls[,'b']) - trueb)/trueb
  res2[,.(b_hat=round((mean(b)-trueb)/trueb*100,4)),by=.(model,n,m)] # test for bias

}

simulate_it_1 <- function(niter=2000,beta=-9999,method_list=c('permF','obrien','roast','GA','gee'),n_array=-99
  # simulate FRED15 (Cole et al. 2015)
  # result statistics are for eudaimonia but hedonia is kept to look at correlation in estimates when effect i
  # do_power - makes the eudaimonia effect equal to beta
  # if beta==-9999 then beta is set to the value estimated by ols using FRED15
  # if n_array=-9999 then n is set the value of FRED15
  # if m_array=-9999 then m is set to the value of FRED15


  # get the empirical data
  fn <- 'cole2_clean.txt'
  dt <- read_file(fn,year=2015)
  dt[,zhedonia:=scale(zhedonia)]
  dt[,zeudaimonia:=scale(zeudaimonia)]
  dt <- contrast_coefficients(dt)  # convert to CTRA response
  xcols <- get_xcols()
  ycols <- c(pro_inflam_genes(year=2015),antibody_genes(),ifn_genes())
  zcols <- 'zeudaimonia'
```

```
dt[,male:=as.integer(as.character(male))]
dt[,white:=as.integer(as.character(white))]
dt[,alcohol:=as.integer(as.character(alcohol))]
dt[,smoke:=as.integer(as.character(smoke))]
X <- dt[,.SD,.SDcols=xcols]
Y <- scale(dt[,.SD,.SDcols=ycols])
dt <- cbind(X,Y)
Ry <- cor(Y)
Rx <- cor(X)
# get mean and variance of expression levels of hedonia and eudaimonia
form <- formula(paste('Y',paste(xcols,collapse='+'),sep='~'))
fitmv <- lm(form, data=dt)
b.hedm <- coefficients(fitmv)['zhedonia',]
b.eudm <- coefficients(fitmv)['zeudaimonia',]
if(beta==-9999){beta.mu <- abs(mean(b.eudm))}
# beta.mu <- 0.06716244
beta.sd <- sd(b.eudm)

n_data <- nrow(dt)
m_data <- ncol(Y)
if(n_array[1]==-9999){n_array <- n_data}
if(m_array[1]==-9999){m_array <- m_data}
param_matrix <- expand.grid(n_array, m_array)
colnames(param_matrix) <- c('n','m')

#output file
code <- sample(LETTERS,4,replace=TRUE)
if(do_power==TRUE){error_type='typeII'}else{error_type='typeI'}
fn_out <- paste('gsa_sim_1',error_type,paste(code,collapse=''),'txt',sep='.')

res <- data.table(NULL)
for(experiment in 1:nrow(param_matrix)){
  n <- param_matrix[experiment,'n']
  m <- param_matrix[experiment,'m']
  N <- m*n
  tvalue <- numeric(m)
  rycols <- paste('Y',1:m,sep='')

  for(iter in 1:niter){

    #  simulated vector of causal effects of eudaimonia on the m expression levels
    # re-center and scale so that the coefficients have precisely the mean and sd specified
    beta_vec <- rnorm(m,mean=beta.mu,sd=beta.sd)
    beta_vec <- (scale(beta_vec))[,1]*beta.sd + beta.mu

    X <- rmvnorm(n=n,sigma=Rx)
    colnames(X) <- xcols
    gene_inc <- sample(1:nrow(Ry),m) # subsample the genes
    E <- rmvnorm(n=n,sigma=Ry[gene_inc,gene_inc]) # matrix of expression levels for m genes
    # make Y a function of eudaimonia if do_power==TRUE otherwise Y=E
    if(do_power==TRUE){Y <- X[,'zeudaimonia']%*%t(beta_vec) + t(matrix(sqrt(1-beta_vec^2),nrow=m,ncol=n))*E}[illegible]
    # scale so that true values have precise specification
    X <- scale(X)
    Y <- scale(Y)
    colnames(Y) <- rycols
    rdt <- data.table(cbind(X,Y))
    rdt[,subject:=factor(.I)]
    # wide to long for GEE/GLS
    dtlong <- melt(rdt,id.vars=c('subject',xcols),variable.name='gene',value.name='expression')
```

```r
dtlong[,gene:=factor(gene)]
dtlong <- orderBy(~subject + gene, dtlong)
# fit ols
X.dm <- cbind(rep(1,n),X)
fit.ols <- lm.fit(X.dm,Y)
b.ols <- mean(fit.ols$coefficients[zcols,])
b.hed <- mean(fit.ols$coefficients['zhedonia',])
if('ols' %in% method_list){
  # not fast code using lm.fit - could use the code in obrien.fit to do this
  form <- formula(paste('expression~',paste(c('gene',xcols),collapse='+'),sep=''))
  fit <- summary(lm(form, data=dtlong)) # used for both .ols and .roast below
  prob <- fit$coefficients['zeudaimonia','Pr(>|t|)']
  res <- rbind(res,data.table(n=n,m=m,model='ols',b=b.ols, prob=prob,b.hed=b.hed))
}
if('obrien' %in% method_list){
  # get R, the correlation among Y conditional on all X but zeudaimonia
  obrien_table <- obrien.fit(rdt,xcols,rycols,zcols)
  prob <- obrien_table[,p]
  res <- rbind(res,data.table(n=n,m=m,model='obrien',b=b.ols, prob=prob,b.hed=b.hed))
}
if('permt' %in% method_list){ # permutation t
  prob <- permutation_t.fit(rdt,xcols=xcols,ycols=rycols,zcols='zeudaimonia',method='t',perms=perms, wri
  res <- rbind(res,data.table(n=n,m=m,model='permt',b=b.ols, prob=prob,b.hed=b.hed))
}
if('R2' %in% method_list){ # permutation t
  prob <- permutation_t.fit(rdt,xcols=xcols,ycols=rycols,zcols='zeudaimonia',method='R2',perms=perms, wr
  res <- rbind(res,data.table(n=n,m=m,model='R2',b=b.ols, prob=prob,b.hed=b.hed))
}
if('permF' %in% method_list){ # permutation t
  prob <- permutation_F.fit(rdt,xcols=xcols,ycols=rycols,zcols='zeudaimonia',method='resid',perms=perms,
  res <- rbind(res,data.table(n=n,m=m,model='permF',b=b.ols, prob=prob,b.hed=b.hed))
}
if('gls' %in% method_list){
  form <- formula(paste('expression~',paste(c('gene',xcols),collapse='+'),sep=''))
  fit.gls <- gls(form, data=dtlong, method='ML',correlation=corCompSymm(form = ~ 1 | subject), weights=v
  # save gls fits
  b.gls <- summary(fit.gls)$tTable['zeudaimonia','Value']
  b.hed <- summary(fit.gls)$tTable['zhedonia','Value']
  prob <- summary(fit.gls)$tTable['zeudaimonia','p-value']
  res <- rbind(res,data.table(n=n,m=m,model='gls',b=b.gls, prob=prob,b.hed=b.hed))
}
if('gls.un' %in% method_list){
  form <- formula(paste('expression~',paste(c('gene',xcols),collapse='+'),sep=''))
  fit.gls <- gls(form, data=dtlong, method='ML', correlation=corSymm(form = ~ 1 | subject), weights=varI
  # save gls fits
  b.gls <- summary(fit.gls)$tTable['zeudaimonia','Value']
  b.hed <- summary(fit.gls)$tTable['zhedonia','Value']
  prob <- summary(fit.gls)$tTable['zeudaimonia','p-value']
  res <- rbind(res,data.table(n=n,m=m,model='gls.un',b=b.gls, prob=prob,b.hed=b.hed))
}
if('gee' %in% method_list){
  # gee
  form <- formula(paste('expression~',paste(c('gene',xcols),collapse='+'),sep=''))
  fit.geeglm <- geeglm(form, family=gaussian, data=dtlong,id=subject,waves=gene, corstr='exchangeable',
  b.gee <- summary(fit.geeglm)$coefficients['zeudaimonia','Estimate']
  b.hed <- summary(fit.geeglm)$coefficients['zhedonia','Estimate']
  prob <- summary(fit.geeglm)$coefficients['zeudaimonia','Pr(>|W|)']
  se.gee <- summary(fit.geeglm)$coefficients['zeudaimonia','Std.err']
  res <- rbind(res,data.table(n=n,m=m,model='gee',b=b.gee, prob=prob,b.hed=b.hed))
```

```
    }
    if('roast' %in% method_list){
      # Roast format
      Yt <- t(Y) # genes as rows
      form <- formula(paste('~',paste(xcols,collapse='+'),sep=''))
      design <- model.matrix(form, data=rdt)
      eudaimonia <- which(colnames(design)=='zeudaimonia')
      roast_res <- roast(y=Yt,design=design,contrast=eudaimonia, nrot=perms)
      prob <- roast_res$p.value['UpOrDown','P.Value']
      res <- rbind(res,data.table(n=n,m=m,model='roast',b=b.ols, prob=prob,b.hed=b.hed))
    }
    if('GA' %in% method_list){ # globalAncova
      Yt <- t(Y) # genes as rows
      form.full <- formula(paste('~',paste(xcols,collapse='+'),sep=''))
      model.dat <- rdt[, .SD, .SDcols=xcols]
      prob <- GlobalAncova(Yt, form.full, model.dat=model.dat, test.terms='zeudaimonia', method='permutation
      res <- rbind(res,data.table(n=n,m=m,model='GA',b=b.ols, prob=prob,b.hed=b.hed))
    }
    if(write_it==TRUE){
      write.table(res,fn_out,sep='\t',quote=FALSE,row.names=FALSE)
    }
  }
 }
 return(res)
}
```

2.3 Script for Generalized GSA methods

```
# script of GSA methods
# Jeffrey A. Walker
# August 22, 2016

library(data.table)

ols.fit <- function(dt,xcols,ycols,zcols,scale_it=TRUE,boot=TRUE,perms=2000,all=FALSE){
  # returns ols estimate and bootstrap SE
  # for parametric SE use Obrien?
  # if all=TRUE then return matrix with rows=perms
  n <- nrow(dt)
  m <- length(ycols)
  p <- length(xcols)
  rows <- 1:n
  b_mat <- matrix(NA,nrow=perms,ncol=length(zcols))
  colnames(b_mat) <- zcols
  for(iter in 1:perms){
    Y <- data.matrix(dt[rows,.SD,.SDcols=ycols])
    if(scale_it==TRUE){
      Y <- scale(Y)
      X1 <- data.matrix(dt[rows, .SD, .SDcols=setdiff(xcols,zcols)])
      X2 <- scale(data.matrix(dt[rows, .SD, .SDcols=zcols]))
      X.dm <- cbind(rep(1,n),X1,X2)
    }else{
      X.dm <- cbind(rep(1,n),data.matrix(dt[rows, .SD, .SDcols=xcols]))  # design matrix
      }
    fit <- lm.fit(X.dm,Y)
    b_mat[iter,] <- apply(fit$coefficients[zcols,],1,mean)
    rows <- sample(1:n,replace=TRUE)
  }
```

```
  b_table <- data.table(
    Ind.Var = zcols,
    Estimate = b_mat[1,],
    SE = apply(b_mat,2,sd)
    )
  b_table[,t:=Estimate/SE]
  b_table[,prob:=2*pt(abs(t),df=n-p-1,lower.tail = FALSE)] # conservative df, upper end should be n*m-p-1
  if(all==TRUE){
    return(b_mat)
  }else{
    return(b_table)
  }
}

ols.fit.long <- function(dt,xcols,ycols,zcols,scale_it=TRUE,boot=TRUE,perms=2000){
  # same as ols.fit but using long instead of wide format. Ouch!
  # microbenchmark results using default settings
  #Unit: seconds
  #expr      min       lq     mean   median       uq      max neval
  #ols.fit(dt, xcols, ycols, zcols) 11.50812 11.50812 11.50812 11.50812 11.50812 11.50812     1
  #ols.fit.long(dt, xcols, ycols, zcols) 97.90578 97.90578 97.90578 97.90578 97.90578 97.90578     1

  n <- nrow(dt)
  m <- length(ycols)
  p <- length(xcols)
  rows <- 1:n
  b_mat <- matrix(NA,nrow=perms,ncol=length(zcols))
  colnames(b_mat) <- zcols
  for(iter in 1:perms){
    dts <- dt[rows]
    if(scale_it==TRUE){
      Y <- scale(data.matrix(dt[rows, .SD, .SDcols=ycols]))
      X1 <- data.matrix(dt[rows, .SD, .SDcols=setdiff(xcols,zcols)])
      X2 <- scale(data.matrix(dt[rows, .SD, .SDcols=zcols]))
      dts <- data.table(X1,X2,Y)
    }
    dts[,subject:=factor(.I)]
    dtlong <- melt(dts,id.vars=c('subject',xcols),variable.name='gene',value.name='expression')
    dtlong[,gene:=factor(gene)]
    dtlong <- orderBy(~subject + gene, dtlong)
    form <- formula(paste('expression~',paste(c('gene',xcols),collapse='+'),sep=''))
    Y <- dtlong[,expression]
    X.mm <- model.matrix(form,data=dtlong)
    fit <- lm.fit(X.mm,Y)
    b_mat[iter,] <- coefficients(fit)[zcols]
    rows <- sample(1:n,replace=TRUE)
  }
  b_table <- data.table(
    Ind.Var = zcols,
    Estimate = b_mat[1,],
    SD = apply(b_mat,2,sd)
  )
  b_table[,t:=Estimate/SD]
  b_table[,prob:=2*pt(abs(t),df=n-p-1,lower.tail = FALSE)] # conservative df, upper end should be n*m-p-1
  return(b_table)
}


ols_estimates <- function(dt,xcols,ycols,zcols){
```

```
  # estimates computed both long and wide format to show equivalence
  # if boot==TRUE then return bootstrap se

  # wide format (multivariate)
  Y <- as.matrix(dt[,.SD,.SDcols=ycols])
  form <- formula(paste('Y',paste(xcols,collapse='+'),sep='~'))
  fit.mv <- lm(form, data=dt)
  bhat <- apply(coefficients(fit.mv)[zcols,],1,mean)

  # long format with multiple outcomes (gene expression levels) stacked into single column
  dt[,subject:=factor(.I)]
  dtlong <- melt(dt,id.vars=c('subject',xcols),variable.name='gene',value.name='expression')
  dtlong[,gene:=factor(gene)]
  dtlong <- orderBy(~subject + gene, dtlong)
  form <- formula(paste('expression~',paste(c('gene',xcols),collapse='+'),sep=''))
  fit.long <- lm(form, data=dtlong)
  bhat.long <- coefficients(fit.long)[zcols]

  return(bhat)
}

obrien.fit <- function(dt,xcols,ycols,zcols){
  # O'Brien's 1984 OLS test for continuous Z
  # fast version of obrien using lm.fit
  # requires that I compute standard errors, t, and prob
  # dt is the data.table with the X specified by xcols and Y specified by ycols
  # zcols is the subarray of xcols to test. currently works with only 1 zcol to test
  # this would be easy to fix by setting whole thing in loop and then rbinding results
  i <- 1 # only 1 zcol
  n <- nrow(dt)
  X.dm <- cbind(rep(1,n),data.matrix(dt[, .SD, .SDcols=xcols])) # design matrix
  Xred <- cbind(rep(1,n),data.matrix(dt[, .SD, .SDcols=setdiff(xcols,zcols[i])]))
  Y <- data.matrix(dt[,.SD,.SDcols=ycols])
  m <- length(ycols)
  df <- nrow(dt) - length(xcols) - 1

  # Get residuals from X (so excluding zcols) to find R - the correlation among the outcomes not explained by
  fit <- lm.fit(Xred,Y)
  R <- cor(fit$residuals)

  # fit full model
  XTXI <- solve(t(X.dm)%*%X.dm)
  fit <- lm.fit(X.dm,Y)
  b <- fit$coefficients[zcols[i],]
  e <- fit$residuals
  se <- sqrt(diag((t(e)%*%e)/df)*XTXI[zcols[i],zcols[i]])
  t_value <- b/se
  #coef_table <- data.table(Estimate=b,se=se,t=t_value)
  t_sum <- sum(t_value)

  obrien.b <- mean(b)
  obrien.t <- t_sum/sqrt(sum(R))
  obrien.sd <- obrien.b/obrien.t
  obrien.p <- 2*pt(abs(obrien.t),df=df,lower.tail = FALSE)
  obrien_table <- data.table(zcols=zcols,b=obrien.b,sd=obrien.sd,t=obrien.t,p=obrien.p)
  return(obrien_table)
}

obrien <- function(dt,xcols,ycols,zcols){
```

```
  # O'Brien's 1984 OLS test for continuous Z
  # dt is the data.table with the X specified by xcols and Y specified by ycols
  # zcols is the subarray of xcols to test. currently works with only 1 zcol to test
  i <- 1 # only 1 zcol
  Y <- data.matrix(dt[,.SD,.SDcols=ycols])
  m <- length(ycols)
  n <- nrow(dt)
  df <- nrow(dt) - length(xcols) - 1

  # coefficients (save t-value in addition to coefficients)
  coef <- matrix(0,nrow=m,ncol=4)
  colnames(coef) <- c('Estimate', 'Std. Error', 't value', 'Pr(>|t|)')

  non_zcols <- which(xcols!=zcols)
  # Get residuals from X (so excluding zcols)
  form <- formula(paste('Y',paste(xcols[non_zcols],collapse='+'),sep='~'))
  R <- cor(residuals(lm(form, data=dt)))

  form <- formula(paste('Y',paste(xcols,collapse='+'),sep='~'))
  fitmv <- lm(form, data=dt)
  sum_fit <- summary(fitmv)
  row <- which(row.names(sum_fit[[paste('Response ',ycols[1],sep='')]]$coefficients)==zcols)
  for(j in 1:m){ # save t-values instead of coefficients
    coef[j,] <- sum_fit[[paste('Response ',ycols[j],sep='')]]$coefficients[row, ]
  }
  obrien.b <- mean(coef[,'Estimate'])
  obrien.t <- sum(coef[,'t value'])/sqrt(sum(R))
  obrien.p <- 2*pt(abs(obrien.t),df=df,lower.tail = FALSE)
  obrien_table <- data.table(zcols=zcols,b=obrien.b,t=obrien.t,p=obrien.p)
  return(obrien_table)
}

permutation_t.fit <- function(dt,xcols,ycols,zcols,method='R2',perms=2000, write_it=FALSE,fn){
  # no testing if there are no nuisance covariates
  # methods include different test statistics
    # t - mean t-value over the m responses
    # R2 - Anderson's R^2 squared partial correlation coefficient
    # maxmean - Efron and Tabrishini's maxmean statistic
  # faster fit using lm.fit
  # permutation using Anderson and Robinson 2001
  # dt is a data.table with the X regressors and Y responses
  # xcols are the regressors
  # ycols are the responses
  # zcols is the xcols to return statistics
  # method=resid uses Anderson permutation of residuals
  # method=pred uses GlobalAncova permutation of predictors
  # fn is the file name to write to

  m <- length(ycols)
  Y <- data.matrix(dt[, .SD, .SDcols=ycols])
  X <- cbind(rep(1,nrow(dt)),data.matrix(dt[, .SD, .SDcols=xcols]))
  XTXI <- solve(t(X)%*%X)
  df <- nrow(dt) - length(xcols) - 1

  res_table <- data.table(matrix(0.0,nrow=perms,ncol=length(zcols)))
  setnames(res_table,zcols)
  for(i in 1:length(zcols)){
    # get residuals from covariates
    if(length(xcols)==length(zcols)){ # no nuissance covariate
```

```
    covs <- xcols
  }else{
    covs <- setdiff(xcols, zcols[i])
  }
  X.red <- cbind(rep(1,nrow(dt)),data.matrix(dt[, .SD, .SDcols=covs]))
  fit.obs <- lm.fit(X.red,Y)
  e <- fit.obs$residuals
  yhat <- fit.obs$fitted.values

  Z <- data.matrix(dt[,.SD,.SDcols=zcols[i]])
  fit.obs <- lm.fit(X.red,Z)
  Rz.x <- fit.obs$residuals # residuals of Z on X
  Rz.x.sqr <- Rz.x^2
  rows <- 1:nrow(dt) # observed on first iter and permuted after
  for(iter in 1:perms){
    Y.pi <- yhat + e[rows,] # permuted
    #form <- formula(paste('Y.pi',paste(c(covs,zcols[i]),collapse='+'),sep='~'))
    #fitmv.pi <- lm(form, data=dt)
    #fitmv_sm <- summary(fitmv.pi)
    #fitmv_sm[[paste('Response ',ycols[j],sep='')]]£coefficients[zcols[i], ]

    if(method=='t'){
      fit.obs <- lm.fit(X,Y.pi)
      b.pi <- fit.obs$coefficients[zcols[i],]
      e.pi <- fit.obs$residuals
      se.pi <- sqrt(diag((t(e.pi)%*%e.pi)/df)*XTXI[zcols[i],zcols[i]])
      t_sum <- sum(b.pi/se.pi)
      res_table[iter,(zcols[i]):=t_sum]
    }

    # anderson and robertson partial correlation test statistic
    if(method=='R2'){
      fit.obs <- lm.fit(X.red,Y.pi)
      e.pi <- fit.obs$residuals
      num <- (sum(e.pi*Rz.x))^2
      denom <- sum(e.pi^2)*sum(Rz.x.sqr)
      R.sqr <- num/denom
      res_table[iter,(zcols[i]):=R.sqr]
    }

    # Efron and tabrishini maxmean statistic
    if(method=='maxmean'){
      fit.obs <- lm.fit(X,Y.pi)
      b.pi <- fit.obs$coefficients[zcols[i],]
      b.posbar <- 0
      b.negbar <- 0
      b.pos <- b.pi[b.pi>0]
      if(length(b.pos)>0){b.posbar <- mean(b.pos)}
      b.neg <- b.pi[b.pi<0]
      if(length(b.neg)>0){b.negbar <- abs(mean(b.neg))}
      maxmean <- max(b.posbar,b.negbar)
      res_table[iter,(zcols[i]):=maxmean]
    }

    # permute rows
    rows <- sample(1:nrow(dt))
  }
}
```

```r
  prob <- apply(res_table,2,function(x) length(which(abs(x) >= abs(x[1]))))/perms)
  return(prob)
}

permutation_F.fit <- function(dt,xcols,ycols,zcols,method='resid',perms=2000, write_it=FALSE,fn){
  # permutation_F using lm.fit
  # GlobalAncova Fga statistic but permutation following Anderson and Robinson 2001
  # dt is a data.table with the X regressors and Y responses
  # xcols are the regressors
  # ycols are the responses
  # method is not implemented here but is in the original permutation_F.
  # method=resid uses Anderson permutation of residuals
  # method=pred uses GlobalAncova permutation of predictors
  # fn is the file name to write to
  # notes: the expected association between permuted hedonic score and gene expression is zero so the expected

  p <- length(ycols)
  Y <- data.matrix(dt[, .SD, .SDcols=ycols])

  Fga <- data.table(matrix(0.0,nrow=perms,ncol=length(zcols)))
  setnames(Fga,zcols)
  for(i in 1:length(zcols)){
    # get residuals from covariates
    covs <- setdiff(xcols, zcols[i])
    #form <- formula(paste('Y',paste(covs,collapse='+'),sep='~'))
    #fit.obs <- lm(form, data=dt)
    #e <- residuals(fit.obs)
    #yhat <- predict(fit.obs) # Yhat = aX
    X.full <- cbind(rep(1,nrow(dt)),data.matrix(dt[, .SD, .SDcols=c(covs,zcols[i])]))
    X.red <- cbind(rep(1,nrow(dt)),data.matrix(dt[, .SD, .SDcols=covs]))
    fit.obs <- lm.fit(X.red,Y)
    e <- fit.obs$residuals
    yhat <- fit.obs$fitted.values
    # e = e1 and yhat=yhat1 to xxx decimal place

    rows <- 1:nrow(dt) # observed on first iter and permuted after
    for(iter in 1:perms){
      Y.pi <- yhat + e[rows,] # permuted
      # full
      fitmv.pi <- lm.fit(X.full,Y.pi) # full model
      e.pi <- fitmv.pi$residuals
      rss.full <- sum(e.pi^2)
      # reduced
      fitmv.pi <- lm.fit(X.red,Y.pi) # reduced model
      e.pi <- fitmv.pi$residuals
      rss.red <- sum(e.pi^2)
      Fga[iter,(zcols[i]):=(rss.red-rss.full)/rss.full]

      # permute rows
      rows <- sample(1:nrow(dt))
    }
  }

  prob <- apply(Fga,2,function(x) length(which(x>=x[1]))/perms)
  return(prob)
}
```
